# Development of a necroptosis-related prognostic model for uterine corpus endometrial carcinoma

**DOI:** 10.1038/s41598-024-54651-3

**Published:** 2024-02-21

**Authors:** Qi Zhang, Yongfu Luo, Shiyao Zhang, Qianpeng Huang, Gang Liu

**Affiliations:** 1 Department of General Surgery, Tianjin Medical University General Hospital, Tianjin, 300052 China; 2 Department of General Surgery, Xintian People’s Hospital, Xintian, 425700 Hunan China

**Keywords:** Uterine corpus endometrial carcinoma, Necroptosis, miRNA, Immunotherapy, Nomogram, Endometrial cancer, Genetic databases

## Abstract

Necroptosis is a recently identified caspase-independent form of cell death which plays a significant role in the onset and progression of cancer. MicroRNAs (miRNAs) are vital for the development of uterine corpus endometrial carcinoma (UCEC) because they are an important regulatory component in necroptosis. This study developed a new necroptosis-related miRNAs profile to predict the prognosis of patients with UCEC. The TCGA-UCEC cohort’s RNA sequencing data, consisting of 534 tumor samples and 33 normal samples, was downloaded. Ten differentially expressed miRNAs related to necroptosis were identified. A prediction model for necroptosis-related miRNAs was then created through COX regression and nomograms analysis. Clinical and pathological parameters were integrated to construct a nomogram and evaluate the model. Prognosis-related miRNAs were further used to predict target genes, and functional analysis was conducted to explore the potential mechanisms of these target genes. Subsequently, immune infiltration analysis was performed using transcriptome data to identify immune genes associated with prognosis, and the expression levels of target gene was validated using UCEC tissues. We identified 7 up-regulated miRNAs (hsa-miR-577, hsa-miR-7-5p, hsa-miR-210-3p, hsa-miR-210-5p, hsa-miR-200a-5p, hsa-miR-141-3p, hsa-miR-425-5p) and 3 down-regulated miRNAs (hsa-miR-7-2-3p, hsa-miR-383-5p, hsa-miR-29a-3p). The risk signature was based on univariate and multivariate COX analyses, constructed using 2 independent prognostic factors and miRNAs (hsa-miR-425-5p, hsa-miR-7-5p) associated with necroptosis. Nomograms demonstrated the prognostic value of risk level, age, FIGO stage, and histological type. Kaplan–Meier analysis revealed significant differences in overall survival (OS) outcomes associated with the expression of hsa-miR-425-5p (*P* < 0.001) and hsa-miR-7-5p (*P* = 0.015). Gene ontology (GO) and Kyoto Encyclopedia of Genes and Genomes (KEGG) investigations indicated that these miRNAs play crucial roles in tumor development, metastasis, and prognosis. Immune infiltration analysis showed decreased infiltration of CD8+ T cells, CD8+ T cells, NK cells, and M1 macrophages in normal tissues. Subsequently, a necroptosis-related immune gene significantly associated with prognosis (THRB) was identified, western blot and immunohistochemical staining confirmed the differential expression of THRB in normal endometrial tissues and tumor. Our findings demonstrate a close association between necroptosis and UCEC. The two necroptosis-related miRNAs used in this study may serve as valuable prognostic markers for UCEC patients, and are associated with immune cell infiltration. This suggests that necroptosis may be involved in the development of UCEC through its interaction with immune responses.

## Introduction

Uterine corpus endometrial carcinoma (UCEC) is globally recognized as the sixth most prevalent cancer among women, making it one of the most frequent gynecological malignancies^1^. Notably, early-stage UCEC often lacks obvious clinical manifestations and is frequently diagnosed at advanced stages characterized by aggressive disease progression, leading to unfavorable outcomes^2^. Currently, the standard treatment modalities for UCEC encompass surgical procedures, radiation therapy, chemotherapy, or their combination^3^. Despite these therapeutic advancements, the 5-year survival rate for treated patients ranges from 70 to 80%^4^. Nevertheless, the precise pathophysiology of UCEC remains poorly understood, underscoring the need for novel diagnostic approaches and prognostic assessment strategies to facilitate personalized therapeutic interventions.

MicroRNAs (miRNAs), with a length ranging from 19 to 25 nucleotides, are short non-coding RNAs of endogenous origin. Despite lacking coding potential, miRNAs exert significant influence by binding to tumor-associated genomic regions or susceptible loci within the genome, thereby modulating the expression of tumor suppressor genes and oncogenes. This intricate regulatory mechanism involving miRNAs is implicated in the initiation and progression of diverse human malignancies^5^.

Degterev et al.^6^ discovered that receptor interacting protein kinase-3 (RIPK3), which activates mixed-lineage kinase domain like protein (MLKL), induces regulated necrosis termed necroptosis. This type of cell death is characterized by a phosphorylation signaling pathway controlled by serine/threonine protein kinases 1/3 (RIPK1/RIPK3), sharing some similarities with apoptosis^7,8^. Necroptosis has been implicated in various inflammatory disorders, including inflammatory bowel disease (IBD), atherosclerosis, skin diseases, acute kidney injury, and systemic inflammatory response syndrome (SIRS)^9,10^. Notably, as key players in the tumor microenvironment, miRNAs actively participate in processes such as epithelial-to-mesenchymal transition (EMT), fibroblast secretion, inflammation, survival, gene expression, and stemness. These processes are critical for regulating tumor cell vitality, as well as tumor initiation, progression, and drug resistance^10^. Necroptosis can be triggered by specific tumor microenvironments, and can also modulate the tumor microenvironment through various miRNAs, including miR-7-5p, miR-148a-3p, miR-141-3p, miR-331-3p, among others^11^. Recent studies indicate that tumor cells resistant to apoptosis may still be susceptible to necroptosis pathways^12,13^, suggesting that investigating tumor cell necroptosis and its regulatory mechanisms could provide potential targets for UCEC therapy. In this study, we analyzed the expression profiles of necroptosis-related miRNAs associated with UCEC using RNA-seq data from TCGA-UCEC cohort, and performed immune infiltration analysis to identify novel biomarkers with clinical and therapeutic significance.

## Methods

### Data processing

We obtained miRNA sequencing data from the TCGA database for 550 patients with TCGA-UCEC, as well as transcriptome sequencing data for 559 patients (https://portal.gdc.cancer.gov). Among these, the clinical and pathological data for 16 patients were censored. Hence, this study included miRNA expression data for 534 tumor tissues and 33 normal tissues. Additionally, we analyzed the complete clinical data, including age, location, FIGO stage, histological type, survival outcome, and duration of survival (Table 1).

**Table 1:** Baseline clinicopathologic characteristics of UCEC patients in the TCGA-UCEC cohort.

Variable	TCGA (n = 534)
Age at diagnosis	
≤ 60 years	207
> 60 years	327
Location	
Endometrium	521
Isthmus uteri	3
Corpus uteri	4
Fundus uteri	6
FIGO stage	
I	334
II	49
III	123
IV	28
Histological type	
Endometrioid adenocarcinoma	401
Serous cystadenocarcinoma	133

### Identification of differentially expressed necroptosis-related miRNAs

Based on previous review studies^11^, 21 miRNAs are known to be associated with necroptosis (miR-181-5P, miR-194-5P, miR-29a-3P, miR-16-5P, miR-148a-3P, miR-338-3P, miR-577, miR-7-3p, miR-7-5p, miR-383-3p, miR-383-5p, miR-210-3p, miR-210-5p, miR-15a-3p, miR-15a-5p, miR-223-3P, miR-200a-5P, miR-500a-3P, miR-141-3P, miR-425-5P, miR-425-3p). The RNA expression data from the TCGA-UCEC cohort were processed using the ‘limma’ package in R, and the differentially expressed miRNAs were identified by the ‘limma’ package (http://www.bioconductor.org/) with the screening criteria of False Discovery Rate (FDR) < 0.05 and |Log2 Fold Change (FC)| > 1.

### Development the prognostic model of necroptosis-related miRNAs

All differentially expressed miRNAs related to necroptosis, obtained from the TCGA-UCEC cohort, were included in the association analysis with overall survival (OS). A *P* value of less than 0.05 was set as the cut-off, and univariate Cox regression analysis was employed to generate *P* values and hazard ratios (HRs). Necroptosis-related miRNAs that demonstrated prognostic relevance in univariate Cox regression analysis were retained for multivariate Cox regression analysis. Subsequently, two miRNAs (hsa-miR-425-5p, hsa-miR-7-5p) were included in the risk model. The risk scores were calculated using the following formula: risk score = (0.223 × hsa-miR-425-5p expression) + (0.181 × hsa-miR-7-5p expression). Survival analysis was carried out using Kaplan–Meier curves. Receiver operating characteristic (ROC) curves were utilized to analyze the patient’s 1-year, 3-year, and 5-year overall survival.

### Development the nomogram model

Clinicopathological parameters were extracted from the TCGA dataset, including age, tumor location, FIGO stage and histological type. Univariate and multivariate COX regression analyses were used to analyze the prognostic value of these variables and risk score. COX regression analysis was then used to create a nomogram of prognosis, which was validated using calibration plots.

### Prediction of target genes and functional analysis

Using miRDB (http://www.mirdb.org/), TargetScan (https://www.targetscan.org/) and miRTarBase (https://mirtarbase.cuhk.edu.cn) databases to predict target genes associated with prognostic miRNAs were identified. The predicted genes in all three databases were selected as miRNAs target genes. R was used for gene ontology (GO) and Kyoto Encyclopedia of Genes and Genomes (KEGG) enrichment analysis^14–16^.

### Tumor immune infiltration analysis

The ‘CIBERSORT’ package of the R software was used to assess the proportions of 22 immune cell types in tumor samples, including seven T-cell types, three B-cell types, NK cells and myeloid cells (https://cibersort.stanford.edu/), and the results were saved for subsequent analysis^17^. Wilcoxon rank-sum test in the R software was used to identify differential expression of tumor-infiltrating immune cells (TICs) between different TMB groups (*P* < 0.05).

TIMER2.0 (http://timer.comp-genomics.org/) is a tumor immunity-related database that enables the assessment of the association between gene expression, mutation status, somatic copy number variation, and immune cell types (herein referred to as the “immune association module”)^18^. The immune cells that can be infiltrated include lymphocytes, macrophages, NK cells, and neutrophils, which can effectively predict the prognosis of patients.

### Validation of target gene based on proteomics analysis

The Human Protein Atlas (HPA), which maps all human proteins in cells, tissues and organs by integrating various omics technologies (https://www.proteinatlas.org/), was used to assess protein-based differences between UCEC and normal tissues^19^. We downloaded the histological section images and corresponding information on overexpressed target genes from normal tissues and cancer tissues obtained by immunohistochemistry from HPA.

### Immunohistochemical staining and western blot

Immunohistochemistry (IHC) was employed to localize and quantify the expression of THRB in normal tissue and UCEC FFPE sections. Tissue sections were deparaffinized and rehydrated, followed by antigen retrieval. Endogenous peroxidase activity was quenched, and sections were then incubated with primary antibodies specific to THRB (AF0357, Affinity Biosciences, Jiangsu, China, dilution 1:100). After washing, sections were incubated with secondary antibodies conjugated to an enzyme label. The enzymatic reaction was visualized using a chromogenic substrate, and counterstaining was performed to visualize cellular morphology. Analysis was conducted using light microscopy, allowing for the localization and quantification of THRB proteins within the tissue samples.

Western blot (WB) was performed to analyze THRB expression levels. Tissue samples were lysed and proteins were separated using SDS-PAGE, then transferred to a PVDF membrane. After blocking, the membrane was probed with a primary antibody against THRB (AF0357, Affinity Biosciences, Jiangsu, China) at a dilution of 1:3000, and subsequently with secondary antibodies. Protein bands were visualized using a fluorescence imaging system or chemiluminescence substrate.

### Statistical analysis

All statistical analysis was performed using R version 4.1.1 and attached packages. Chi-square test was used to compare categorical variables, *t*-test for comparison of means, and Wilcoxon rank-sum test for other comparisons. Kaplan–Meier survival curves and log-rank test were used for survival analysis. *P* < 0.05 indicated statistical significance.

## Results

### Differential expression of necroptosis-related miRNAs in UCEC

**Figure 1:**
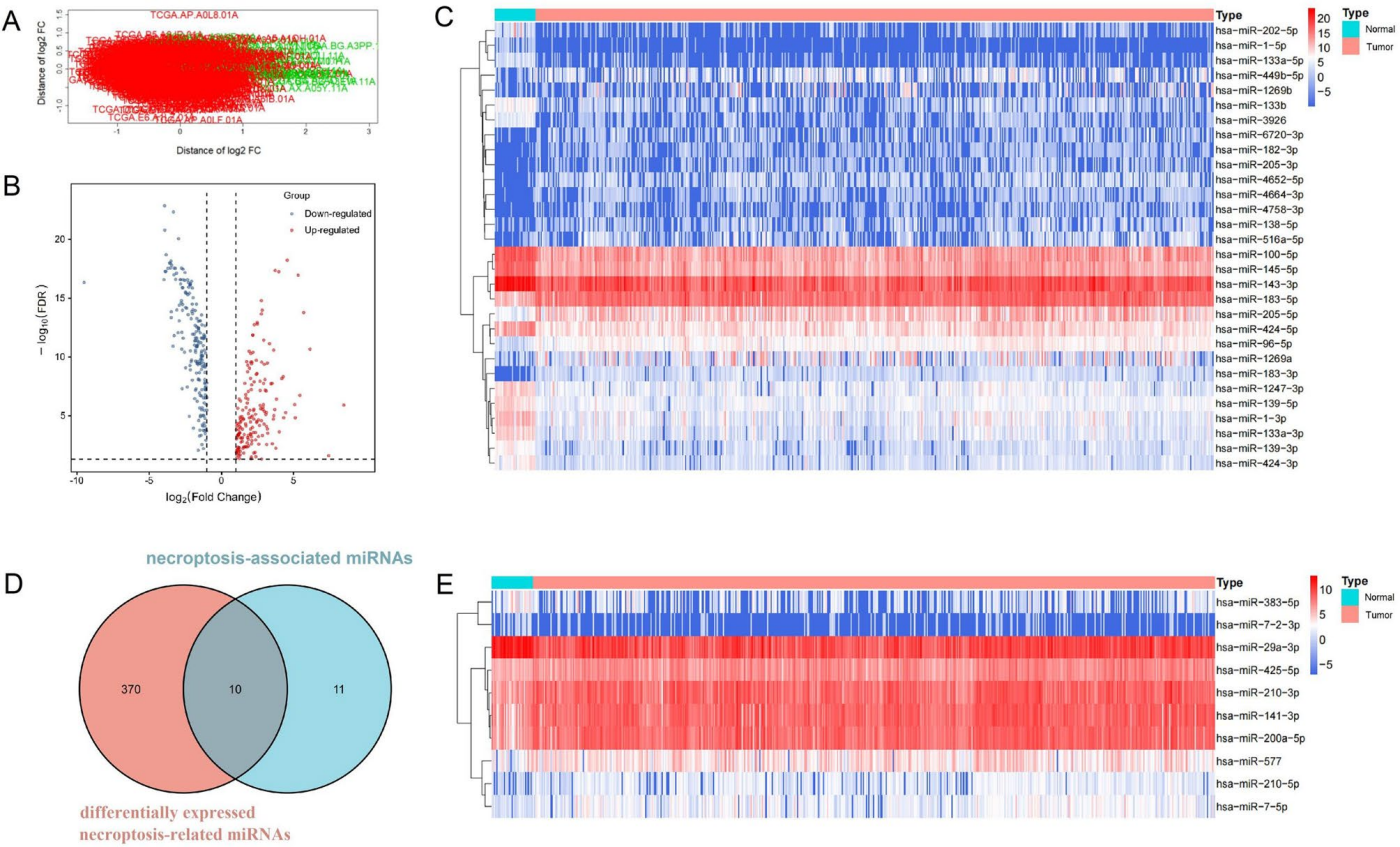
Analysis of necroptosis-associated miRNAs. (**A**) Cluster analysis of normal tissues and tumor tissues. (**B**) A total of 380 differentially expressed miRNAs were identified in tumor tissues, with 177 up-regulated and 203 down-regulated. (**C**) Heatmap illustrating the differences in expression levels between normal tissues and tumor tissues. (**D**) Venn diagram showing 10 differentially expressed miRNAs associated with necroptosis. (**E**) Heatmap displaying the differential expression of necroptosis-associated miRNAs.

We performed differential expression analysis of miRNA sequencing in 33 normal tissues and 534 tumor tissues from the TCGA-UCEC cohort. The clustering analysis in Fig. 1A demonstrated a clear distinction between normal and tumor tissues. Applying the criteria of FDR < 0.05 and absolute Log2 FC > 1, a total of 380 differentially expressed miRNAs were identified in tumor tissues, with 177 upregulated and 203 downregulated (Fig. 1B). The top 30 miRNAs based on differential expression are presented in a heatmap (Fig. 1C). To identify miRNAs associated with necroptosis, we cross-referenced the obtained 380 differentially expressed miRNAs with all necroptosis-related miRNAs from a previous study^11^, yielding 10 differentially expressed miRNAs associated with necroptosis (Fig. 1D). The heatmap of their differential expression is shown in Fig. 1E, indicating that 7 miRNAs were upregulated and 3 miRNAs were downregulated in tumor tissues. Log2 FC, *P* value, and FDR for each miRNA are displayed in Table 2.

**Table 2:** 10 differentially expressed necroptosis-associated miRNAs’ Log2 FC, *P* value and FDR.

Necroptosis-associated miRNA	Log2 FC	*P* Value	FDR
hsa-miR-29a-3p	− 2.286	2.26E−18	4.62E−17
hsa-miR-383-5p	− 2.225	5.31E−06	1.11E−05
hsa-miR-200a-5p	2.764	1.23E−16	1.65E−15
hsa-miR-210-3p	2.765	2.18E−15	2.17E−14
hsa-miR-141-3p	2.169	2.00E−13	1.35E−12
hsa-miR-425-5p	2.274	2.13E−14	1.77E−13
hsa-miR-7-5p	2.673	3.49E−09	1.15E−08
hsa-miR-210-5p	2.088	7.61E−09	2.37E−08
hsa-miR-577	1.355	1.56E−05	3.00E−05
hsa-miR-7-2-3p	− 1.675	2.30E−07	5.79E−07

### Development the prognostic model of necroptosis-related miRNAs

**Figure 2:**
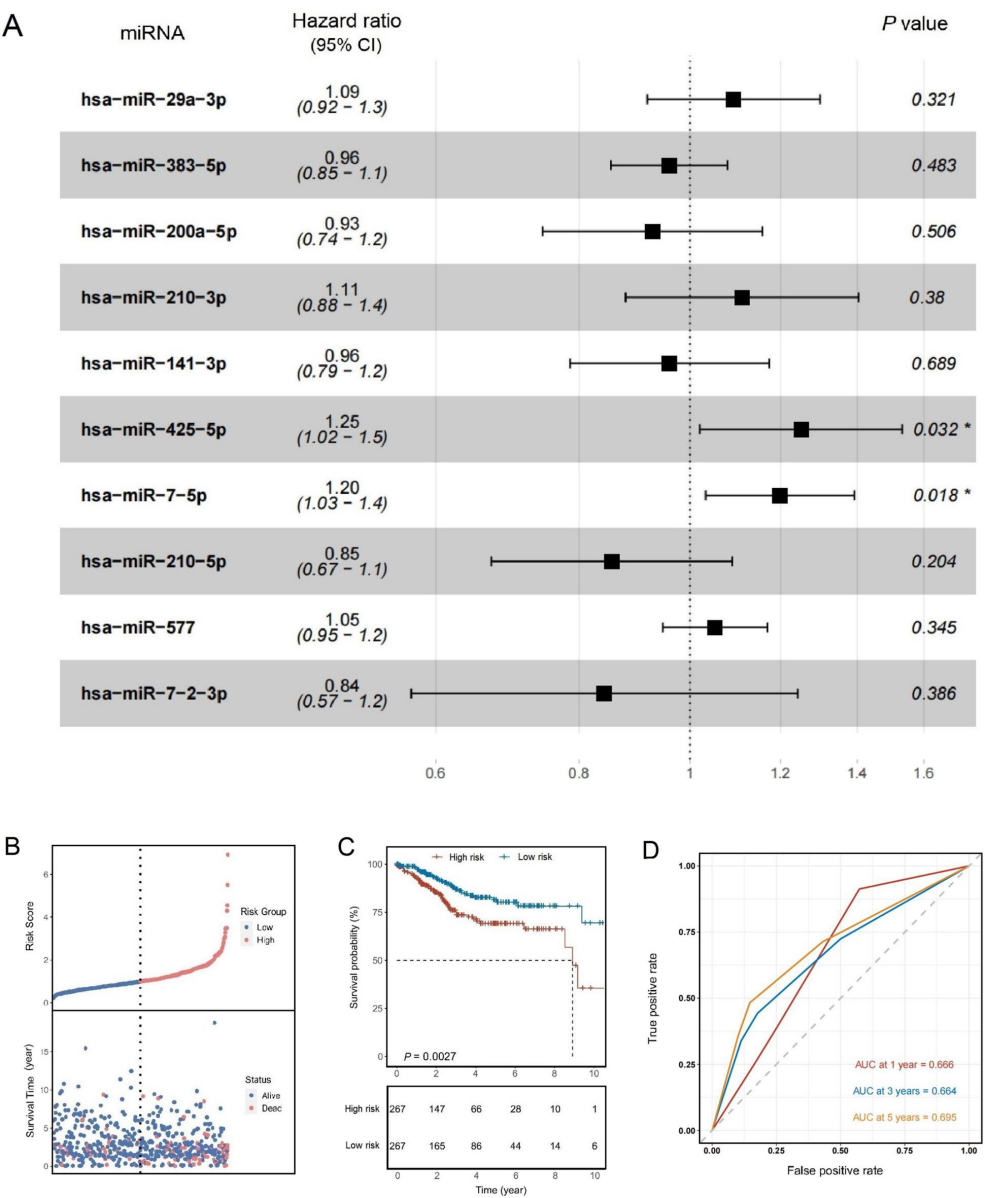
Development of a prognostic model using necroptosis-related miRNAs. (**A**) Multivariate Cox analysis revealed that hsa-miR-425-5p and hsa-miR-7-5p, two miRNAs associated with necroptosis, were significantly correlated with prognosis (*P* < 0.05). (**B**) The 534 patients were classified into low-risk and high-risk groups based on the median risk score. (**C**) Kaplan–Meier survival curves illustrating the survival differences between different risk levels. (**D**) ROC curves demonstrating the prognostic value of the risk score.

Based on the previous analysis, differentially expressed miRNAs associated with necroptosis were identified. Considering their potential prognostic value, univariate and multivariate Cox analyses were performed, which revealed a significant association between hsa-miR-425-5p and hsa-miR-7-5p, two necroptosis-related miRNAs, and prognosis (*P* < 0.05) (Table 3, Fig. 2A). The impact of the prognostic model constructed by these two miRNAs on patient survival was assessed using Kaplan–Meier curves. The 534 patients were divided into low- and high-risk groups based on the median risk score (Fig. 2B), with the high-risk group displaying poorer prognosis (*P* = 0.003) (Fig. 2C). Receiver operating characteristic (ROC) curve analysis evaluated the sensitivity and specificity of the prognostic model, with the AUC for the 1-year, 3-year, and 5-year survival rates being 0.666, 0.664, and 0.695, respectively (Fig. 2D), indicating good predictive value of this feature.

**Table 3:** Univariate and multivariate Cox regression analyses of necroptosis-associated miRNAs.

Necroptosis-associated miRNA	Univariate COX regression		Multivariate COX regression	
	HR	*P* value	HR	*P* value
hsa-miR-29a-3p	1.181	0.019	1.092	0.321
hsa-miR-383-5p	0.990	0.865	0.959	0.483
hsa-miR-200a-5p	1.105	0.208	0.928	0.506
hsa-miR-210-3p	1.132	0.097	1.111	0.380
hsa-miR-141-3p	1.105	0.211	0.960	0.689
hsa-miR-425-5p	1.363	< 0.001	1.250	0.032
hsa-miR-7-5p	1.259	< 0.001	1.198	0.018
hsa-miR-210-5p	1.073	0.368	0.854	0.204
hsa-miR-577	1.069	0.201	1.052	0.345
hsa-miR-7-2-3p	0.855	0.431	0.842	0.386

### Development of a prognostic nomogram

**Figure 3:**
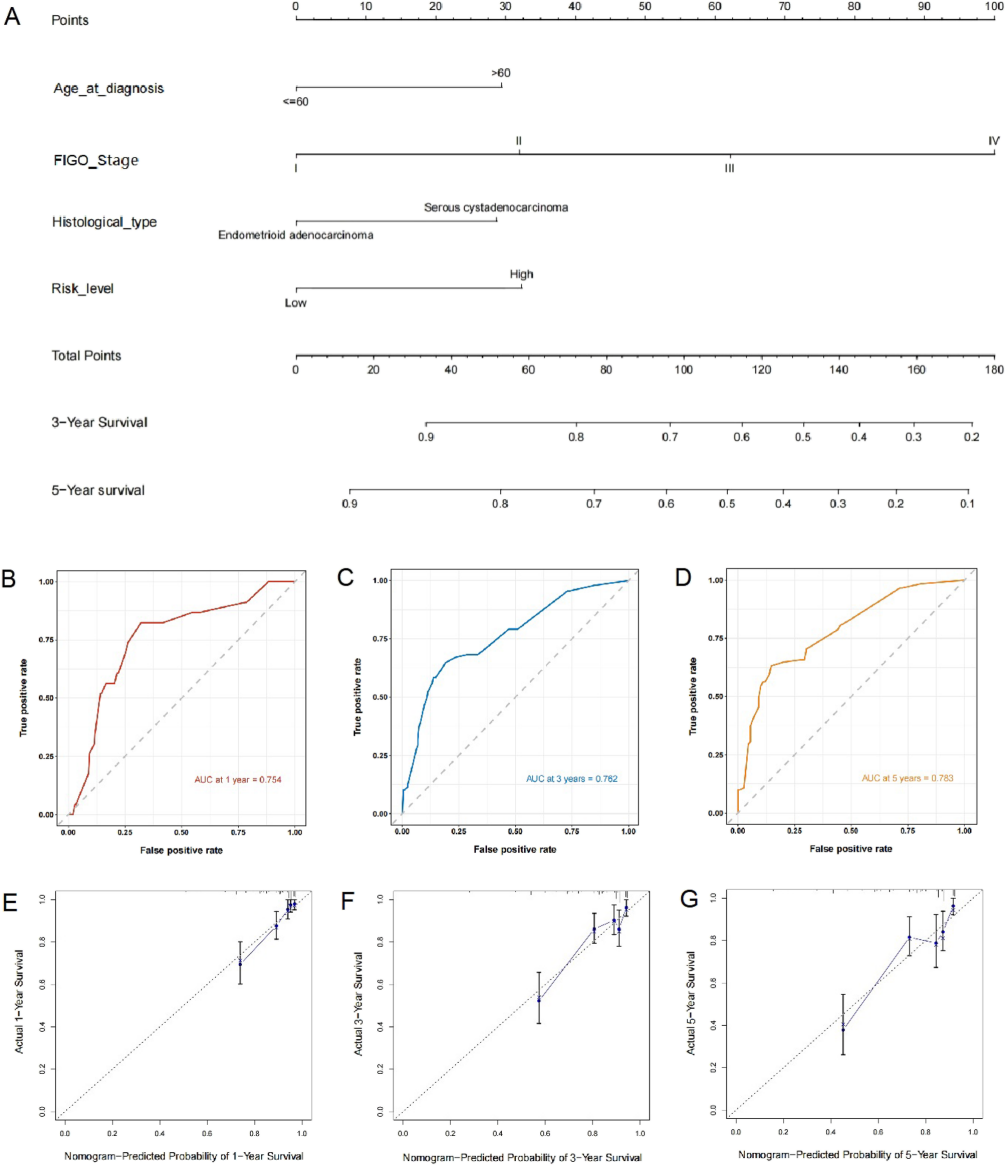
Construction of a prognostic nomogram model. (**A**) Nomogram predicting the 3- and 5-year overall survival (OS). ROC curves demonstrating the prognostic value of the nomogram model at (**B**) 1-year, (**C**) 3-year, and (**D**) 5-year OS. Calibration plots of the nomogram to predict the probability of (**E**) 1-year, (**F**) 3-year, and (**G**) 5-year OS.

Univariate and multivariate Cox regression analyses were conducted to determine whether the risk score served as an independent predictor of patient outcomes. In the univariate Cox regression analysis, the risk score, age, FIGO stage, and histological type exhibited an association with the prognosis in UCEC patients. Following adjustment for potential confounding variables, the multivariate Cox regression analysis revealed that the risk score, age, FIGO stage, and histological type remained independent predictors of prognosis in UCEC patients (Table 4). Rosters were created for 3-year and 5-year overall survival (OS) based on the risk score, age, FIGO staging, and histological type (Fig. 3A). The variable score was determined by the intersection of the vertical line with the point axis associated with each variable, and the total risk score was the sum of the scores for each variable. These scores were used to predict the OS for each patient. The C-index was determined as 0.753. Receiver operating characteristic (ROC) curve analysis was used to evaluate the sensitivity and specificity of the prognostic model, yielding an AUC of 0.754, 0.762, and 0.783 for the 1-year, 3-year, and 5-year survival rates, respectively (Fig. 3B–D). Finally, the calibration graphs visually demonstrated good agreement between the predicted and actual outcomes at 1, 3, and 5 years (Fig. 3E–G).

**Table 4:** Univariate and multivariate Cox regression analyses of risk factors in UCEC.

Variable	Univariate COX regression		Multivariate COX regression	
	HR	*P* value	HR	*P* value
Age at diagnosis	1.856	0.006	1.660	0.033
FIGO stage	3.749	< 0.001	3.268	< 0.001
Histological type	1.867	0.006	1.647	0.035
Risk level	1.877	0.005	1.698	0.029

### Identification of prognostic necroptosis-related miRNAs

**Figure 4:**
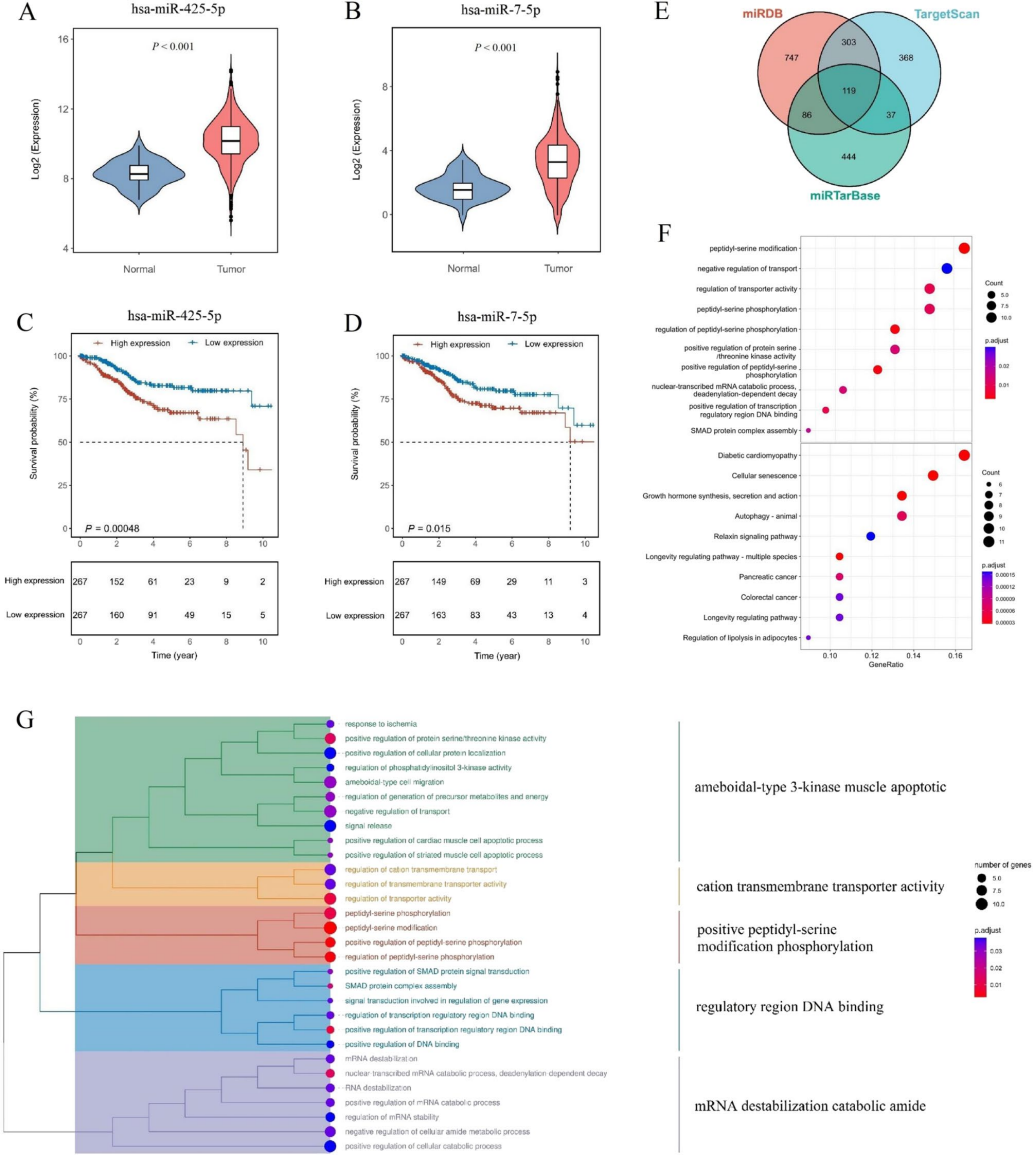
Identification of prognostic necroptosis-related miRNAs. In tumor tissues, hsa-miR-425-5p (**A**) and hsa-miR-7-5p (**B**) demonstrated high expression levels. Patients with high expression levels of hsa-miR-425-5p (**C**) and hsa-miR-7-5p (**D**) exhibited poorer prognosis. (**E**) Prediction of target genes for these 2 key miRNAs was performed using three online databases. Enrichment analysis of gene ontology (GO) and Kyoto Encyclopedia of Genes and Genomes (KEGG) for the target genes is presented in (**F**,**G**).

The expression levels of hsa-miR-425-5p and hsa-miR-7-5p in tumor tissues were analyzed, and it was found that both miRNAs were highly expressed in tumor tissues (Fig. 4A,B). To further elucidate the correlation between necroptosis-related miRNAs and prognosis, survival analysis was performed for each miRNA in the signature. The results showed that both miRNAs used in the signature were significantly associated with prognosis (*P* < 0.05), with patients displaying high expression levels of hsa-miR-425-5p and hsa-miR-7-5p in tumor tissues showing a poorer prognosis (Fig. 4C,D). Prediction of target genes for these two key miRNAs was carried out using three online databases, resulting in a total of 2852 target genes (850 for hsa-miR-425-5p and 2002 for hsa-miR-7-5p). After removing duplicates and considering the intersection of the three databases, 119 target genes were identified (Fig. 4E, Supplementary Table 1). The 119 target genes were subsequently subjected to GO and KEGG analyses (Fig. 4F,G). The results of the GO and KEGG enrichment analyses revealed that the target genes were enriched in pathways such as ameboidal-type 3-kinase muscle apoptotic, mRNA destabilization, carcinogenesis disease, catabolic amide, negative regulation of transport, positive regulation of protein serine/threonine kinase activity, and cellular senescence.

### Immune infiltration of TICs

**Figure 5:**
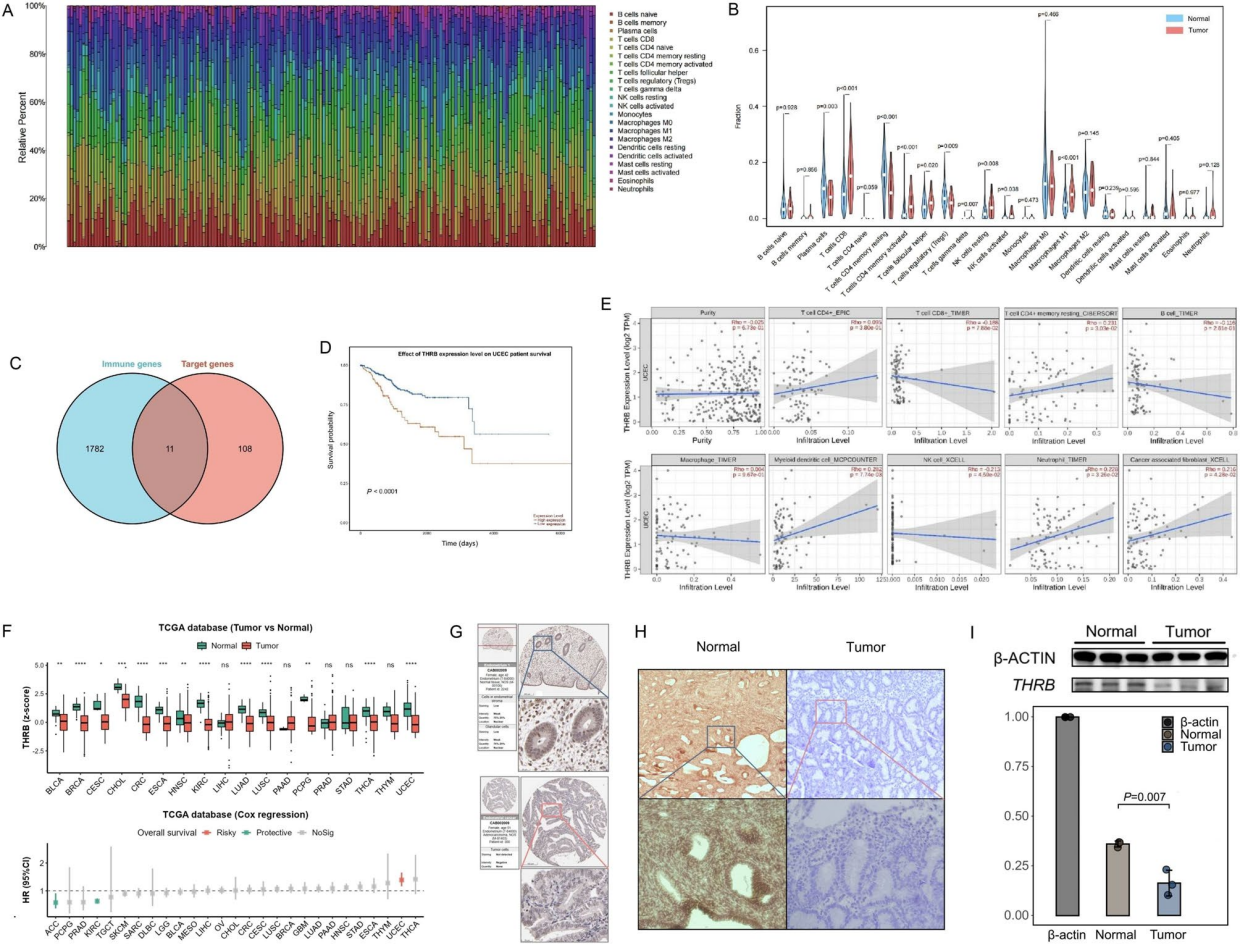
Immune Infiltration analyse of TICs and identification of prognostic target gene in UCEC. (**A**) Bar chart depicted the composition of immune cells in each sample from the TCGA-UCEC cohort. (**B**) Violin plot illustrated the differential infiltration of 22 types of TICs. (**C**) Venn diagram presented 11 immune-related target genes. (**D**) Survival analysis demonstrated a significant impact of the THRB gene on prognosis (*P* < 0.001). (**E**) Analysis of the TIMER2.0 database revealed the relationship between the THRB gene and tumor purity as well as immune cell infiltration levels. (**F**) Pan-cancer data analysis from TCGA showed the expression levels of THRB in various tumor tissues and its impact on prognosis. (**G**) Immunohistochemistry results from HPA database exhibited differential expression of THRB in normal and tumor tissues. The results of both IHC (**H**) and WB (**I**) showed a decrease in the expression level of THRB in tumor tissues.

We analyzed the differences in immune cell infiltration levels between normal and tumor tissues. Figure 5A displays the proportions of immune cells in each sample from the TCGA-UCEC cohort, while the violin plot shows the differences in 22 Tumor-Infiltrating Immune Cells (TICs) (Fig. 5B). In tumor tissues, the percentages of CD8+ T cells, T cells CD4 memory activated, T cells gamma delta, NK cells, and Macrophages M1 were higher, while the percentages of Plasma cells, Tregs, and T cells CD4 memory resting were lower (*P* < 0.05).

### Identification of prognostic target gene in UCEC

To identify novel genes associated with immune infiltration in tumor tissues, we intersected the previously obtained 119 target genes (Fig. 4E) with the Immunology Database and Analysis Portal (ImmPort) (https://www.dev.immport.org/home) to obtain a list of all human immune-related genes (Fig. 5C). We identified 11 immune-related target genes (Supplementary Table 2), and using Kaplan–Meier survival analysis, we found that the THRB gene had a significant impact on patient prognosis (*P* < 0.001) (Fig. 5D). Subsequently, we used the TIMER 2.0 database to analyze the relationship between THRB expression and tumor purity and immune cell infiltration levels. We found that THRB expression was not significantly correlated with tumor purity but showed a positive correlation with immune cell infiltration levels (Fig. 5E).

The analysis of pan-cancer data from TCGA reveals that THRB is expressed at lower levels in various tumor tissues, including UCEC, compared to normal tissues (Fig. 5F). Furthermore, THRB emerges as an independent prognostic risk factor in UCEC, consistent with the findings in Fig. 5D. Immunohistochemistry results from the HPA database indicated differential expression of THRB in normal and tumor tissues (Fig. 5G). Subsequently, we conducted validation experiments using surgical specimens from UCEC patients treated at our center, and both the IHC (Fig. 5H) and WB (*P* = 0.007) (Fig. 5I) results demonstrated a decrease in THRB expression levels in tumor tissues. Additionally, the original WB image of THRB has been included as Supplementary Fig. 1.

## Discussion

Endometrial carcinoma, also known as uterine corpus endometrial cancer (UCEC), is a malignant neoplasm characterized by abnormal proliferation of endometrial tissue. It represents a significant global health burden, accounting for 7% of all newly diagnosed cancers in 2022 and contributing to 4% of cancer-related deaths^20^. The incidence and mortality rates of UCEC have exhibited an upward trend in recent years. Given its predilection for perimenopausal and postmenopausal women, the aging population is expected to further contribute to the growing burden of UCEC. UCEC is a heterogeneous malignancy, featuring a propensity for both local and distant recurrence. Prognosis is profoundly influenced by histological subtype and clinical stage, impeding the development of effective screening and therapeutic strategies. Although early-stage UCEC patients who undergo surgical intervention followed by adjuvant radiation and chemotherapy exhibit favorable 5-year survival rates, therapeutic options remain limited for advanced-stage or recurrent metastatic disease, consequently resulting in a bleak prognosis^21^. Thus, there is a pressing need to delineate novel therapeutic targets and discover innovative biomarkers for UCEC.

MiRNAs play a pivotal role in tumorigenesis and can be categorized as either oncogenic miRNAs (oncomiRs) or tumor-suppressor miRNAs. OncomiRs, which function as oncogenes, are upregulated in tumor cells, whereas tumor-suppressor miRNAs, acting as tumor suppressor genes, are downregulated in tumor cells^22^. The expression profiles of miRNAs display unique patterns across various cancers, including breast cancer, lung cancer, chronic lymphocytic leukemia, and UCEC^23,24^. In endometrial tumor tissue, dysregulated miRNAs are likely involved in cellular processes such as proliferation, differentiation, apoptosis, and carcinogenesis^25,26^. Programmed cell death is a highly regulated and orderly form of cellular demise encompassing various modes, such as apoptosis, autophagy, necroptosis, ferroptosis, and pyroptosis. The induction of programmed cell death in tumor cells plays a crucial role in cancer treatment. Necroptosis has emerged as a dual player in cancer, as key regulators of necroptosis can either independently or synergistically promote cancer metastasis and progression. Conversely, when apoptotic pathways are impaired, necroptosis can serve as a protective mechanism, preventing tumor development and metastasis.

In this study, we conducted an analysis of the expression levels of 21 miRNAs associated with necroptosis. Among them, hsa-miR-425-5p and hsa-miR-7-5p were identified as prognostically independent risk miRNAs, exhibiting significantly elevated expression in UCEC tumors compared to normal tissue counterparts. To enhance the accuracy of overall survival (OS) prediction in UCEC patients, we developed a robust prognostic model and risk scoring system based on these two miRNAs. This scoring system serves as a valuable tool for assessing and predicting the prognosis of UCEC patients. The prognostic model considers the independent influence of the risk score, alongside other clinical factors including age, FIGO stage, and histological type. Furthermore, we constructed a novel nomogram by integrating the risk score and clinical parameters, facilitating the personalized prognostication of UCEC patients. The efficiency and reliability of this nomogram were validated using thorough calibration curve analysis.

In the present study, we conducted comprehensive univariate and multivariate Cox analyses, identifying hsa-miR-425-5p and hsa-miR-7-5p as independent prognostic risk factors in UCEC. We observed significantly elevated expression levels of these necroptosis-related miRNAs in UCEC tumor tissues, and Kaplan–Meier survival analysis revealed their significant impact on overall survival (OS) outcomes of UCEC patients. Previous investigations have shed light on the pivotal roles of these two miRNAs in diverse malignancies. For instance, Liu et al. reported that hsa-miR-425-5p might enhance tumor occurrence and metastasis by activating the adherens junction protein pathway mediated by ctnnd1^27^. Urh et al. highlighted significant differences in hsa-miR-425-5p expression between adenoma, colorectal cancer, and metastatic colorectal cancer, suggesting its potential involvement in tumorigenesis and metastasis^28^. Hsa-miR-425-5p has also been implicated in stemness and cisplatin resistance in laryngeal cancer cells^29^. Furthermore, its downregulation has been associated with poor prognosis in ovarian cancer^30^. Another investigation demonstrated that hsa-miR-425-5p inactivates the Wnt/β-catenin signaling pathway, consequently reducing the expression of MALAT1 and TUG1 in osteosarcoma, thereby inhibiting tumor progression^31^. Additionally, miR-7-5p has been implicated in the inhibition of tumor metastasis by targeting NOVA2 in non-small cell lung cancer (NSCLC)^32^, RELA in breast cancer^33^, and HOXB13 in esophageal squamous cell carcinoma^34^. In rhabdomyosarcoma (RMS), miR-7 exerts an anti-tumor effect by targeting mitochondrial proteins SLC25A37 and TIMM50, ultimately inducing necrosis^35^. Moreover, SLC25A37 is significantly overexpressed in patients with high cytotoxicity^36^, while TIM50 deficiency suppresses tumor cell growth and induces apoptosis in breast cancer^37^. Based on the findings of these previous investigations, the two miRNAs identified in our study, which markedly impact the survival of UCEC patients, are also closely associated with prognosis in other malignancies.

Moreover, this investigation delved into the functional characterization of potential target genes regulated by differentially expressed necrosis-associated miRNAs. Notably, KEGG pathway analysis revealed a significant enrichment of these target genes in signaling pathways implicated in carcinogenesis, mRNA instability, and other related diseases. Subsequently, identification of a potential necrosis-associated immune-related gene emerged. Immune infiltration analysis substantiated a close association between THRB and various immune cell populations, highlighting its potential role in UCEC development. THRB has been extensively studied in the context of tumorigenesis, with most investigations demonstrating decreased THRB mRNA expression^38^. In conclusion, this study establishes a comprehensive prognostic model that accurately predicts the clinical outcomes of UCEC patients, considering crucial factors such as overall survival, age, FIGO stage, and histological type. Furthermore, the functional assessment of potential target genes controlled by necrosis-related miRNAs was successfully carried out, elucidating their involvement in diverse pathways encompassing carcinogenesis and mRNA instability through comprehensive KEGG pathway analysis.

Overall, the findings of this study underscore a potential link between necrosis and the pathogenesis and advancement of UCEC. Nevertheless, it is important to acknowledge certain limitations within this investigation. Firstly, the use of only the TCGA-UCEC dataset restricts the generalizability of the results, emphasizing the necessity for additional datasets to validate these analyses. Moreover, the absence of experimental validation regarding the expression, functionality, and mechanistic actions of the identified miRNAs hinders a comprehensive understanding of their roles. Therefore, further in vitro and in vivo investigations are warranted to substantiate the clinical observations presented herein.

## Conclusions

This study successfully identified and characterized novel miRNA markers associated with necroptosis, providing valuable insights into the prognosis of patients with UCEC. The results obtained from this study contribute to the application of precision medicine in clinical diagnosis and therapeutic strategies.

### Supplementary Information

Supplementary Information 1.

Supplementary Information 2.

Supplementary Information 3.

## Data Availability

The datasets generated during the current study are available in the TCGA database [https://portal.gdc.cancer.gov/projects/TCGA-UCEC].
