# Supplementary material for: Development of a necroptosis-related prognostic model for uterine corpus endometrial carcinoma
Source: Sci Rep. 2024 Feb 21;14:4257. doi: 10.1038/s41598-024-54651-3 (PMC10881509; doi:10.1038/s41598-024-54651-3)
Supplement: Supplementary file 1 — Supplementary Information 1. [file 41598_2024_54651_MOESM1_ESM.docx]

**C_index.R**

install.packages("rms")

dir="E:\\necroptosis\\R_clinical\\5_C_index"

setwd(dir)

library(survival)

library(rms)

TCGA<-read.table("input.txt",header=T,sep="\t") #??为?约???????

TCGA$Age_at_diagnosis<-factor(TCGA$Age_at_diagnosis,labels=c("<=60",">60"))

TCGA$Stage<-factor(TCGA$Stage,labels=c("I","II","III","IV"))

TCGA$Histological_type<-factor(TCGA$Histological_type,labels=c("Endometrioid adenocarcinoma","Serous cystadenocarcinoma"))

TCGA$Risk_level<-factor(TCGA$Risk_level,labels=c("Low","High"))

fmla1 <- as.formula(Surv(Survival_time,Status) ~Age_at_diagnosis + Stage + Histological_type + Risk_level)

cox2 <- coxph(fmla1,data=TCGA)

summary(cox2)

**Calibration.R**

dir="E:\\necroptosis\\R_clinical\\6_Calibration"

setwd(dir)

library(survival)

library(rms)

TCGA<-read.table("input.txt",header=T,sep="\t") #??Ϊ?Լ???????

TCGA$Age_at_diagnosis<-factor(TCGA$Age_at_diagnosis,labels=c("<=60",">60"))

TCGA$Stage<-factor(TCGA$Stage,labels=c("I","II","III","IV"))

TCGA$Histological_type<-factor(TCGA$Histological_type,labels=c("Endometrioid adenocarcinoma","Serous cystadenocarcinoma"))

TCGA$Risk_level<-factor(TCGA$Risk_level,labels=c("Low","High"))

#1-year

cox1 <- cph(Surv(Survival_time,Status) ~ Age_at_diagnosis + Stage + Histological_type + Risk_level,surv=T,x=T, y=T,time.inc = 1*365*2,data=TCGA)

cal <- calibrate(cox1, cmethod="KM", method="boot", u=1*365*2, m= 100, B=400)

pdf("calibrate1.pdf")

plot(cal,lwd=2,lty=1,errbar.col="black",xlim = c(0,1),ylim = c(0,1),xlab ="Nomogram-Predicted Probability of 1-Year Survival",ylab="Actual 1-Year Survival",col="blue",sub=F)

mtext("")

box(lwd = 0.5)

abline(0,1,lty = 3,lwd = 2,col = "black")

dev.off()

#3-year

cox1 <- cph(Surv(Survival_time,Status) ~ Age_at_diagnosis + Stage + Histological_type + Risk_level,surv=T,x=T, y=T,time.inc = 1*365*3,data=TCGA)

cal <- calibrate(cox1, cmethod="KM", method="boot", u=1*365*3, m= 100, B=400)

pdf("calibrate3.pdf")

plot(cal,lwd=2,lty=1,errbar.col="black",xlim = c(0,1),ylim = c(0,1),xlab ="Nomogram-Predicted Probability of 3-Year Survival",ylab="Actual 3-Year Survival",col="blue",sub=F)

mtext("")

box(lwd = 0.5)

abline(0,1,lty = 3,lwd = 2,col = "black")

dev.off()

#5-year

cox2 <- cph(Surv(Survival_time,Status) ~ Age_at_diagnosis + Stage + Histological_type + Risk_level,surv=T,x=T, y=T,time.inc = 1*365*5,data=TCGA)

cal <- calibrate(cox2, cmethod="KM", method="boot", u=1*365*5, m= 100, B=400)

pdf("calibrate5.pdf")

plot(cal,lwd=2,lty=1,errbar.col="black",xlim = c(0,1),ylim = c(0,1),xlab ="Nomogram-Predicted Probability of 5-Year Survival",ylab="Actual 5-Year Survival",col="blue",sub=F)

mtext("")

box(lwd = 0.5)

abline(0,1,lty = 3,lwd = 2,col = "black")

dev.off()

**diff.R**

library(limma)

library(pheatmap)

setwd("E:\\necroptosis\\R_miRNA\\1_diff")

inputFile="miRNA.txt"

fdrFilter=0.05

logFCfilter=1

conNum=33

treatNum=545

outTab=data.frame()

grade=c(rep(1,conNum),rep(2,treatNum))

rt=read.table(inputFile,sep="\t",header=T,check.names=F)

rt=as.matrix(rt)

library("limma")

setwd("C:\\Users\\Co1on\\Desktop\\R\\19.CIBERSORT")

expFile="symbol.txt"

rt=read.table(expFile,sep="\t",header=T,check.names=F)

rt=as.matrix(rt)

rownames(rt)=rt[,1]

exp=rt[,2:ncol(rt)]

dimnames=list(rownames(exp),colnames(exp))

data=matrix(as.numeric(as.matrix(exp)),nrow=nrow(exp),dimnames=dimnames)

data=avereps(data)

if(grepl("-",colnames(data)[ncol(data)])){

group=sapply(strsplit(colnames(data),"\\-"),"[",4)

group=sapply(strsplit(group,""),"[",1)

group=gsub("2","1",group)

data=data[,group==0]

}

data=data[rowMeans(data)>0,]

v <-voom(data, plot = F, save.plot = F)

out=v$E

out=rbind(ID=colnames(out),out)

write.table(out,file="uniq.symbol.txt",sep="\t",quote=F,col.names=F)

source("TMBimmune19.CIBERSORT.R")

results=CIBERSORT("ref.txt", "uniq.symbol.txt", perm=100, QN=TRUE)

rownames(rt)=rt[,1]

exp=rt[,2:ncol(rt)]

dimnames=list(rownames(exp),colnames(exp))

data=matrix(as.numeric(as.matrix(exp)),nrow=nrow(exp),dimnames=dimnames)

data=avereps(data)

data=data[rowMeans(data)>0.2,]

for(i in row.names(data)){

geneName=unlist(strsplit(i,"\\|",))[1]

geneName=gsub("\\/", "_", geneName)

rt=rbind(expression=data[i,],grade=grade)

rt=as.matrix(t(rt))

wilcoxTest<-wilcox.test(expression ~ grade, data=rt)

conGeneMeans=mean(data[i,1:conNum])

treatGeneMeans=mean(data[i,(conNum+1):ncol(data)])

logFC=log2(treatGeneMeans)-log2(conGeneMeans)

pvalue=wilcoxTest$p.value

conMed=median(data[i,1:conNum])

treatMed=median(data[i,(conNum+1):ncol(data)])

diffMed=treatMed-conMed

if( ((logFC>0) & (diffMed>0)) | ((logFC<0) & (diffMed<0)) ){

outTab=rbind(outTab,cbind(gene=i,conMean=conGeneMeans,treatMean=treatGeneMeans,logFC=logFC,pValue=pvalue))

}

}

pValue=outTab[,"pValue"]

fdr=p.adjust(as.numeric(as.vector(pValue)),method="fdr")

outTab=cbind(outTab,fdr=fdr)

write.table(outTab,file="all.xls",sep="\t",row.names=F,quote=F)

outDiff=outTab[( abs(as.numeric(as.vector(outTab$logFC)))>logFCfilter & as.numeric(as.vector(outTab$fdr))<fdrFilter),]

write.table(outDiff,file="diff.xls",sep="\t",row.names=F,quote=F)

write.table(outDiff,file="diff.txt",sep="\t",row.names=F,quote=F)

heatmap=rbind(ID=colnames(data[as.vector(outDiff[,1]),]),data[as.vector(outDiff[,1]),])

write.table(heatmap,file="diffGeneExp.txt",sep="\t",col.names=F,quote=F)

geneNum=15

diffSig=outDiff[order(as.numeric(as.vector(outDiff$logFC))),]

diffGeneName=as.vector(diffSig[,1])

diffLength=length(diffGeneName)

hmGene=c()

if(diffLength>(geneNum*2) ){

hmGene=diffGeneName[c(1:geneNum,(diffLength-geneNum+1):diffLength)]

}else{

hmGene=diffGeneName

}

hmExp=data[hmGene,]

hmExp=log2(hmExp+0.001)

Type=c(rep("Normal",conNum),rep("Tumor",treatNum))

names(Type)=colnames(data)

Type=as.data.frame(Type)

pdf(file="heatmap.pdf",height=5,width=20)

pheatmap(hmExp,

annotation=Type,

color = colorRampPalette(c("royalblue", "white", "red"))(50),

cluster_cols =F,

show_colnames = F,

fontsize = 18,

fontsize_row=16,

fontsize_col=18)

dev.off()

**immune19.run.R**

library("limma")

setwd("C:\\Users\\Co1on\\Desktop\\R\\19.CIBERSORT")

expFile="symbol.txt"

rt=read.table(expFile,sep="\t",header=T,check.names=F)

rt=as.matrix(rt)

rownames(rt)=rt[,1]

exp=rt[,2:ncol(rt)]

dimnames=list(rownames(exp),colnames(exp))

data=matrix(as.numeric(as.matrix(exp)),nrow=nrow(exp),dimnames=dimnames)

data=avereps(data)

if(grepl("-",colnames(data)[ncol(data)])){

group=sapply(strsplit(colnames(data),"\\-"),"[",4)

group=sapply(strsplit(group,""),"[",1)

group=gsub("2","1",group)

data=data[,group==0]

}

data=data[rowMeans(data)>0,]

v <-voom(data, plot = F, save.plot = F)

out=v$E

out=rbind(ID=colnames(out),out)

write.table(out,file="uniq.symbol.txt",sep="\t",quote=F,col.names=F)

source("TMBimmune19.CIBERSORT.R")

results=CIBERSORT("ref.txt", "uniq.symbol.txt", perm=100, QN=TRUE)

**immune19.CIBERSORT.R**

#' CIBERSORT R script v1.03

#' Note: Signature matrix construction is not currently available; use java version for full functionality.

#' Author: Aaron M. Newman, Stanford University (amnewman@stanford.edu)

#' Requirements:

#' R v3.0 or later. (dependencies below might not work properly with earlier versions)

#' install.packages('e1071')

#' install.pacakges('parallel')

#' install.packages('preprocessCore')

#' if preprocessCore is not available in the repositories you have selected, run the following:

#' source("http://bioconductor.org/biocLite.R")

#' biocLite("preprocessCore")

#' Windows users using the R GUI may need to Run as Administrator to install or update packages.

#' This script uses 3 parallel processes. Since Windows does not support forking, this script will run

#' single-threaded in Windows.

#'

#' Usage:

#' Navigate to directory containing R script

#'

#' In R:

#' source('CIBERSORT.R')

#' results <- CIBERSORT('sig_matrix_file.txt','mixture_file.txt', perm, QN)

#'

#' Options:

#' i) perm = No. permutations; set to >=100 to calculate p-values (default = 0)

#' ii) QN = Quantile normalization of input mixture (default = TRUE)

#'

#' Input: signature matrix and mixture file, formatted as specified at http://cibersort.stanford.edu/tutorial.php

#' Output: matrix object containing all results and tabular data written to disk 'CIBERSORT-Results.txt'

#' License: http://cibersort.stanford.edu/CIBERSORT_License.txt

#' Core algorithm

#' @param X cell-specific gene expression

#' @param y mixed expression per sample

#' @export

CoreAlg <- function(X, y){

#try different values of nu

svn_itor <- 3

res <- function(i){

if(i==1){nus <- 0.25}

if(i==2){nus <- 0.5}

if(i==3){nus <- 0.75}

model<-svm(X,y,type="nu-regression",kernel="linear",nu=nus,scale=F)

model

}

if(Sys.info()['sysname'] == 'Windows') out <- mclapply(1:svn_itor, res, mc.cores=1) else

out <- mclapply(1:svn_itor, res, mc.cores=svn_itor)

nusvm <- rep(0,svn_itor)

corrv <- rep(0,svn_itor)

#do cibersort

t <- 1

while(t <= svn_itor) {

weights = t(out[[t]]$coefs) %*% out[[t]]$SV

weights[which(weights<0)]<-0

w<-weights/sum(weights)

u <- sweep(X,MARGIN=2,w,'*')

k <- apply(u, 1, sum)

nusvm[t] <- sqrt((mean((k - y)^2)))

corrv[t] <- cor(k, y)

t <- t + 1

}

#pick best model

rmses <- nusvm

mn <- which.min(rmses)

model <- out[[mn]]

#get and normalize coefficients

q <- t(model$coefs) %*% model$SV

q[which(q<0)]<-0

w <- (q/sum(q))

mix_rmse <- rmses[mn]

mix_r <- corrv[mn]

newList <- list("w" = w, "mix_rmse" = mix_rmse, "mix_r" = mix_r)

}

#' do permutations

#' @param perm Number of permutations

#' @param X cell-specific gene expression

#' @param y mixed expression per sample

#' @export

doPerm <- function(perm, X, Y){

itor <- 1

Ylist <- as.list(data.matrix(Y))

dist <- matrix()

while(itor <= perm){

#print(itor)

#random mixture

yr <- as.numeric(Ylist[sample(length(Ylist),dim(X)[1])])

#standardize mixture

yr <- (yr - mean(yr)) / sd(yr)

#run CIBERSORT core algorithm

result <- CoreAlg(X, yr)

mix_r <- result$mix_r

#store correlation

if(itor == 1) {dist <- mix_r}

else {dist <- rbind(dist, mix_r)}

itor <- itor + 1

}

newList <- list("dist" = dist)

}

#' Main functions

#' @param sig_matrix file path to gene expression from isolated cells

#' @param mixture_file heterogenous mixed expression

#' @param perm Number of permutations

#' @param QN Perform quantile normalization or not (TRUE/FALSE)

#' @export

CIBERSORT <- function(sig_matrix, mixture_file, perm=0, QN=TRUE){

library(e1071)

library(parallel)

library(preprocessCore)

#read in data

X <- read.table(sig_matrix,header=T,sep="\t",row.names=1,check.names=F)

Y <- read.table(mixture_file, header=T, sep="\t", row.names=1,check.names=F)

X <- data.matrix(X)

Y <- data.matrix(Y)

#order

X <- X[order(rownames(X)),]

Y <- Y[order(rownames(Y)),]

P <- perm #number of permutations

#anti-log if max < 50 in mixture file

if(max(Y) < 50) {Y <- 2^Y}

#quantile normalization of mixture file

if(QN == TRUE){

tmpc <- colnames(Y)

tmpr <- rownames(Y)

Y <- normalize.quantiles(Y)

colnames(Y) <- tmpc

rownames(Y) <- tmpr

}

#intersect genes

Xgns <- row.names(X)

Ygns <- row.names(Y)

YintX <- Ygns %in% Xgns

Y <- Y[YintX,]

XintY <- Xgns %in% row.names(Y)

X <- X[XintY,]

#standardize sig matrix

X <- (X - mean(X)) / sd(as.vector(X))

#empirical null distribution of correlation coefficients

if(P > 0) {nulldist <- sort(doPerm(P, X, Y)$dist)}

#print(nulldist)

header <- c('Mixture',colnames(X),"P-value","Correlation","RMSE")

#print(header)

output <- matrix()

itor <- 1

mixtures <- dim(Y)[2]

pval <- 9999

#iterate through mixtures

while(itor <= mixtures){

y <- Y[,itor]

#standardize mixture

y <- (y - mean(y)) / sd(y)

#run SVR core algorithm

result <- CoreAlg(X, y)

#get results

w <- result$w

mix_r <- result$mix_r

mix_rmse <- result$mix_rmse

#calculate p-value

if(P > 0) {pval <- 1 - (which.min(abs(nulldist - mix_r)) / length(nulldist))}

#print output

out <- c(colnames(Y)[itor],w,pval,mix_r,mix_rmse)

if(itor == 1) {output <- out}

else {output <- rbind(output, out)}

itor <- itor + 1

}

#save results

write.table(rbind(header,output), file="CIBERSORT-Results.txt", sep="\t", row.names=F, col.names=F, quote=F)

#return matrix object containing all results

obj <- rbind(header,output)

obj <- obj[,-1]

obj <- obj[-1,]

obj <- matrix(as.numeric(unlist(obj)),nrow=nrow(obj))

rownames(obj) <- colnames(Y)

colnames(obj) <- c(colnames(X),"P-value","Correlation","RMSE")

obj

}

**immunebarplot.R**

setwd("C:\\Users\\Co1on\\Desktop\\R\\20.barplot")

input="CIBERSORT-Results.txt"

outpdf="barplot.pdf"

pFilter=0.05

immune=read.table("CIBERSORT-Results.txt",sep="\t",header=T,row.names=1,check.names=F)

immune=immune[immune[,"P-value"]<pFilter,]

immune=as.matrix(immune[,1:(ncol(immune)-3)])

data=t(immune)

col=rainbow(nrow(data),s=0.7,v=0.7)

pdf(outpdf,height=10,width=22)

par(las=1,mar=c(8,5,4,16),mgp=c(3,0.1,0),cex.axis=1.5)

a1 = barplot(data,col=col,yaxt="n",ylab="Relative Percent",xaxt="n",cex.lab=1.8)

a2=axis(2,tick=F,labels=F)

axis(2,a2,paste0(a2*100,"%"))

axis(1,a1,labels=F)

par(srt=60,xpd=T);text(a1,-0.02,colnames(data),adj=1,cex=0.6);par(srt=0)

ytick2 = cumsum(data[,ncol(data)])

ytick1 = c(0,ytick2[-length(ytick2)])

legend(par('usr')[2]*0.98,par('usr')[4],legend=rownames(data),col=col,pch=15,bty="n",cex=1.3)

dev.off()

**immunevenn.R**

install.packages("VennDiagram")

library(VennDiagram)

setwd("C:\\Users\\Co1on\\Desktop\\R\\22.immuneGene")

geneList=list()

rt=read.table("immune.txt",header=F,sep="\t",check.names=F)

geneList[["Immune"]]=as.vector(rt[,1])

#??ȡ?????????ļ?

rt=read.table("diff.txt",header=T,sep="\t",check.names=F,row.names=1)

#logFC2=rt[abs(rt$logFC)>2,]

#geneList[["|logFC|>2"]]=row.names(logFC2)

logFC0.5=rt[abs(rt$logFC)>0.5,]

geneList[["|logFC|>0.5"]]=row.names(logFC0.5)

venn.plot=venn.diagram(geneList,filename=NULL,main.cex = 2,

fill=c("darkblue", "darkgreen"),cat.cex=1)

pdf(file="venn.pdf",width=8,height=8)

grid.draw(venn.plot)

dev.off()

upGenes=Reduce(intersect,geneList)

write.table(file="intersectGenes.txt",upGenes,sep="\t",quote=F,row.names=F,col.names=F)

**immunevioplot.R**

library(vioplot)

library(limma)

pFilter=0.05

setwd("C:\\Users\\Co1on\\Desktop\\R\\21.vioplot")

immune=read.table("CIBERSORT-Results.txt",sep="\t",header=T,row.names=1,check.names=F)

immune=immune[immune[,"P-value"]<pFilter,]

immune=as.matrix(immune[,1:(ncol(immune)-3)])

rownames(immune)=gsub("(.*?)\\-(.*?)\\-(.*?)\\-(.*?)\\-.*","\\1\\-\\2\\-\\3",rownames(immune))

immune=avereps(immune)

tmb=read.table("TMB.txt",sep="\t",header=T,check.names=F,row.names=1)

tmb=as.matrix(tmb)

row.names(tmb)=gsub("(.*?)\\-(.*?)\\-(.*?)\\-(.*?)\\-.*","\\1\\-\\2\\-\\3",row.names(tmb))

tmb=avereps(tmb)

lowTmb=tmb[tmb[,"TMB"]<=median(tmb[,"TMB"]),]

highTmb=tmb[tmb[,"TMB"]>median(tmb[,"TMB"]),]

lowTmbName=names(lowTmb)

highTmbName=names(highTmb)

lowTmbImm=intersect(row.names(immune),lowTmbName)

highTmbImm=intersect(row.names(immune),highTmbName)

rt=rbind(immune[lowTmbImm,],immune[highTmbImm,])

lowTmbNum=length(lowTmbImm)

highTmbNum=length(highTmbImm)

pdf("vioplot.pdf",height=8,width=13)

par(las=1,mar=c(10,6,3,3))

x=c(1:ncol(rt))

y=c(1:ncol(rt))

plot(x,y,

xlim=c(0,63),ylim=c(min(rt),max(rt)+0.02),

main="",xlab="", ylab="Fraction",

pch=21,

col="white",

xaxt="n")

for(i in 1:ncol(rt)){

if(sd(rt[1:lowTmbNum,i])==0){

rt[1,i]=0.001

}

if(sd(rt[(lowTmbNum+1):(lowTmbNum+highTmbNum),i])==0){

rt[(lowTmbNum+1),i]=0.001

}

lowTmbData=rt[1:lowTmbNum,i]

highTmbData=rt[(lowTmbNum+1):(lowTmbNum+highTmbNum),i]

vioplot(lowTmbData,at=3*(i-1),lty=1,add = T,col = 'greelightskyblue vioplot(highTmbData,at=3*(i-1)+1,lty=1,add = T,col = 'red'lightcoral wilcoxTest=wilcox.test(lowTmbData,highTmbData)

p=wilcoxTest$p.value

mx=max(c(lowTmbData,highTmbData))

lines(c(x=3*(i-1)+0.2,x=3*(i-1)+0.8),c(mx,mx))

text(x=3*(i-1)+0.5, y=mx+0.02, labels=ifelse(p<0.001, paste0("p<0.001"), paste0("p=",sprintf("%.03f",p))), cex = 0.8)

}

text(seq(1,64,3),-0.05,xpd = NA,labels=colnames(rt),cex = 1,srt = 45,pos=2)

dev.off()

**univariateCox.R**

library(survival)

dir="E:\\necroptosis\\R_miRNA\\3_UnivariateCox"

setwd(dir)

inputfile="merger_data.txt"

miRNA<-read.table(inputfile,header=T,sep="\t",row.names = 1,check.names = F)

miRNAEXP=log2(miRNA[,3:ncol(miRNA)]+1)

miRNA=cbind(miRNA[,1:2],miRNAEXP)

coxR=data.frame()

coxf<-function(x){

fmla1 <- as.formula(Surv(survival_time,status)~miRNA[,x])

mycox <- coxph(fmla1,data=miRNA)

}

for(a in colnames(miRNA[,3:ncol(miRNA)])){

mycox=coxf(a)

coxResult = summary(mycox)

coxR=rbind(coxR,cbind(miRNAname=a,HR=coxResult$coefficients[,"exp(coef)"],

P=coxResult$coefficients[,"Pr(>|z|)"]))

}

write.table(coxR,"coxResult.txt",sep="\t",row.names=F,quote=F)

library(survminer)

pdf("forest1.pdf",12,8)

ggforest(mycox,fontsize = 1)

dev.off()

**multivariateCox.R**

library(survival)

dir="E:\\necroptosis\\R_miRNA\\03MultivariateCox"

setwd(dir)

inputfile="merger_data.txt"

miRNA<-read.table(inputfile,header=T,sep="\t",row.names = 1,check.names = F,stringsAsFactors = F)

miRNAEXP=log2(miRNA[,3:ncol(miRNA)]+1)

miRNA=cbind(miRNA[,1:2],miRNAEXP)

miRNA[,"survival_time"]=miRNA[,"survival_time"]/365

fmla1 <- as.formula(Surv(survival_time,status)~.)

mycox <- coxph(fmla1,data=miRNA)

risk_score<-predict(mycox,type="risk",newdata=miRNA)

risk_level<-as.factor(ifelse(risk_score>median(risk_score),"High","Low"))

write.table(cbind(id=rownames(cbind(miRNA[,1:2],risk_score,risk_level)),cbind(miRNA[,1:2],risk_score,risk_level)),"risk_score.txt",sep="\t",quote=F,row.names=F)

summary(mycox)

library(survminer)

pdf("forest1.pdf",10,7)

ggforest(mycox,fontsize = 1)

dev.off()

**nomogram_Cox.R**

install.packages('car')

install.packages('rms')

install.packages('pROC')

install.packages('timeROC')

install.packages("devtools")

devtools::install_github('yikeshu0611/ggDCA')

options(unzip ='internal')

rm(list = ls())

library(car)

library(rms)

library(pROC)

library(timeROC)

library(ggDCA)

data_dir <- choose.dir(default = "E:\\SEER\\data_dir", caption = "")

output_dir <- choose.dir(default = "E:\\SEER\\data_dir", caption = "")

training_dataset_path <- choose.files(default = data_dir, caption = "",

multi = TRUE, filters = Filters,

index = nrow(Filters))

training_dataset<- read.csv(training_dataset_path, header = TRUE,sep="\t", stringsAsFactors=FALSE)

print(paste0("",dim(training_dataset)[1],"； ",dim(training_dataset)[2],""))

validation_dataset_path <- choose.files(default = data_dir, caption = "",

multi = TRUE, filters = Filters,

index = nrow(Filters))

validation_dataset<- read.csv(validation_dataset_path, header = TRUE,sep="\t", stringsAsFactors=FALSE)

print(paste0("",dim(validation_dataset)[1],"； ",dim(validation_dataset)[2]," "))

comparison_nomogram<-function(com_type,training_dataset,validation_dataset,var_name,var_type){

if(com_type=="btw_datasets"){

data_1=training_dataset[,var_name]

data_2=validation_dataset[,var_name]

}else if(com_type=="btw_grp"){

data_1=training_dataset[(training_dataset[,2]==0),var_name]

data_2=training_dataset[(training_dataset[,2]==1),var_name]

}

if(var_type=="continue_type"){

judge_p<-function(p1,p2,p3,tp,zhp){

mark<-0

if(p1<0.05){

mark<-1

}

if(p2<0.05){

mark<-1

}

if(p3<0.05){

mark<-1

}

if(mark==0){

return(tp)

}else{

return(zhp)

}

}

grp_1_ztp<-shapiro.test(data_1)[2][[1]]

grp_2_ztp<-shapiro.test(data_2)[2][[1]]

y_leveneT=c(data_1, data_2)

group_leveneT=as.factor(c(rep(1,length(data_1)), rep(2,length(data_2))))

fcp<-leveneTest(y_leveneT,group_leveneT)[3][[1]][1]

t_testp<-t.test(data_1,data_2,paired=F)[3][[1]]

df = data.frame(y_leveneT,group_leveneT)

zhp<-wilcox.test(y_leveneT~group_leveneT, df)[3][[1]]

finalp<-judge_p(p1=grp_1_ztp, p2=grp_2_ztp, p3=fcp, tp=t_testp, zhp=zhp)

}else if(var_type=="bi_type"){

col_matrix_producer<-function(grp_0_dingxing_data,grp_1_dingxing_data){

vars<-unique(c(names(table(grp_0_dingxing_data)),names(table(grp_1_dingxing_data))))

col_matrix<-matrix(data = 0, nrow = length(vars), ncol = 2, byrow = FALSE,dimnames = NULL)

colnames(col_matrix)<-c("grp_0","grp_1")

rownames(col_matrix)<-vars

for(index_var in 1:length(vars)){

if(!is.na(table(grp_0_dingxing_data)[vars[index_var]][[1]])){

col_matrix[vars[index_var],"grp_0"]<-table(grp_0_dingxing_data)[vars[index_var]][[1]]

}

}

for(index_var in 1:length(vars)){

if(!is.na(table(grp_1_dingxing_data)[vars[index_var]][[1]])){

col_matrix[vars[index_var],"grp_1"]<-table(grp_1_dingxing_data)[vars[index_var]][[1]]

}

}

return(col_matrix)

}

col_matrix_ratio_producer<-function(col_matrix, round_num){

out_table<-col_matrix

for(index_col in 1:ncol(col_matrix)){

sum_col<-sum(col_matrix[,index_col])

for(index_row in 1:nrow(col_matrix)){

ratio<-round(100*col_matrix[index_row,index_col]/sum_col, round_num)

out_table[index_row,index_col]<-paste0(col_matrix[index_row,index_col], " (",ratio,"%)")

}

}

return(out_table)

}

b0 = which(data_1=="NA")

if(length(b0)){

data_1<-data_1[-b0]

}

b1 = which(data_2=="NA")

if(length(b1)){

data_2<-data_2[-b1]

}

col_matrix<-col_matrix_producer(data_1,data_2)

chisqcp<-chisq.test(col_matrix)[3][[1]]

fisherp<- tryCatch(fisher.test(col_matrix)[1][[1]],error=function(e){return("A")} )

if(fisherp=="A"){

fisherp<-fisher.test(col_matrix,simulate.p.value=TRUE)[1][[1]]

}

#fisherp<-fisher.test(col_matrix)[1][[1]]

col_matrix_observed<-chisq.test(col_matrix)$observed

col_matrix_expected<-chisq.test(col_matrix)$expected

round_num<-1

col_matrix_ratio<-col_matrix_ratio_producer(col_matrix, round_num)

sum_N<-sum(col_matrix)

if(any(col_matrix_expected<5) | sum_N<40){

finalp<-fisherp

}else{

finalp<-chisqcp

}

}

return(paste0("",var_name,"",finalp))

}

ddist <- datadist(training_dataset)

options(datadist='ddist')

f_cph <- cph(Surv(OS,Censor) ~ Risk_level,

x=T, y=T, surv=T,

data=training_dataset)

print(f_cph)

ddist <- datadist(training_dataset)

options(datadist='ddist')

colnames(training_dataset)

f_cph <- cph(Surv(OS,Censor) ~ Age+Stage+Risk_level,

x=T, y=T, surv=T,

data=training_dataset)

print(f_cph)

ddist <- datadist(training_dataset)

options(datadist='ddist')

med <- Quantile(f_cph)

surv <- Survival(f_cph)

pdf(paste0(output_dir,"//nomogram.pdf"),width=13, height=10)

plot(nomogram(f_cph, fun=list(function(x) surv(365, x),

function(x) surv(365*3, x),

function(x) surv(365*5, x)),

funlabel=c("1-year Survival Probability",

"3-year Survival Probability",

"5-year Survival Probability"))

)

dev.off()

pred_f_training<-predict(f_cph,training_dataset,type="lp")#!!!type="lp",是他没错

data_table<-data.frame(time=training_dataset[,"OS"],status=training_dataset[,"Censor"],score=pred_f_training)

time_roc_res <- timeROC(

T = data_table$time,

delta = data_table$status,

marker = data_table$score,

cause = 1,

weighting="marginal",

times = c(365, 3*365, 5*365),

ROC = TRUE,

iid = TRUE

)

time_ROC_df <- data.frame(

TP_1year = time_roc_res$TP[, 1],

FP_1year = time_roc_res$FP[, 1],

TP_3year = time_roc_res$TP[, 2],

FP_3year = time_roc_res$FP[, 2],

TP_5year = time_roc_res$TP[, 3],

FP_5year = time_roc_res$FP[, 3]

)

pdf(file=paste(output_dir, "\\ROC_135_year_training.pdf", sep = ""),width=6,height=6)

ggplot(data = time_ROC_df) +

geom_line(aes(x = FP_1year, y = TP_1year), size = 1, color = "#BC3C29FF") +

geom_line(aes(x = FP_3year, y = TP_3year), size = 1, color = "#0072B5FF") +

geom_line(aes(x = FP_5year, y = TP_5year), size = 1, color = "#E18727FF") +

geom_abline(slope = 1, intercept = 0, color = "grey", size = 1, linetype = 2) +

theme_bw() +

annotate("text",

x = 0.75, y = 0.25, size = 4.5,

label = paste0("AUC at 1 year = ", sprintf("%.3f", time_roc_res$AUC[[1]])), color = "#BC3C29FF"

) +

annotate("text",

x = 0.75, y = 0.15, size = 4.5,

label = paste0("AUC at 3 years = ", sprintf("%.3f", time_roc_res$AUC[[2]])), color = "#0072B5FF"

) +

annotate("text",

x = 0.75, y = 0.05, size = 4.5,

label = paste0("AUC at 5 years = ", sprintf("%.3f", time_roc_res$AUC[[3]])), color = "#E18727FF"

) +

labs(x = "False positive rate", y = "True positive rate") +

theme(

axis.text = element_text(face = "bold", size = 11, color = "black"),

axis.title.x = element_text(face = "bold", size = 14, color = "black", margin = margin(c(15, 0, 0, 0))),

axis.title.y = element_text(face = "bold", size = 14, color = "black", margin = margin(c(0, 15, 0, 0)))

)

dev.off()

pdf(file=paste(output_dir, "\\ROC_1_year_trainging.pdf", sep = ""),width=6,height=6)

ggplot(data = time_ROC_df) +

geom_line(aes(x = FP_1year, y = TP_1year), size = 1, color = "#BC3C29FF") +

geom_abline(slope = 1, intercept = 0, color = "grey", size = 1, linetype = 2) +

theme_bw() +

annotate("text",

x = 0.75, y = 0.15, size = 4.5,

label = paste0("AUC at 1 year = ", sprintf("%.3f", time_roc_res$AUC[[1]])), color = "#BC3C29FF"

) +

labs(x = "False positive rate", y = "True positive rate") +

theme(

axis.text = element_text(face = "bold", size = 11, color = "black"),

axis.title.x = element_text(face = "bold", size = 14, color = "black", margin = margin(c(15, 0, 0, 0))),

axis.title.y = element_text(face = "bold", size = 14, color = "black", margin = margin(c(0, 15, 0, 0)))

)

dev.off()

pdf(file=paste(output_dir, "\\ROC_3_year_trainging.pdf", sep = ""),width=6,height=6)

ggplot(data = time_ROC_df) +

geom_line(aes(x = FP_3year, y = TP_3year), size = 1, color = "#0072B5FF") +

geom_abline(slope = 1, intercept = 0, color = "grey", size = 1, linetype = 2) +

theme_bw() +

annotate("text",

x = 0.75, y = 0.15, size = 4.5,

label = paste0("AUC at 3 years = ", sprintf("%.3f", time_roc_res$AUC[[2]])), color = "#0072B5FF"

) +

labs(x = "False positive rate", y = "True positive rate") +

theme(

axis.text = element_text(face = "bold", size = 11, color = "black"),

axis.title.x = element_text(face = "bold", size = 14, color = "black", margin = margin(c(15, 0, 0, 0))),

axis.title.y = element_text(face = "bold", size = 14, color = "black", margin = margin(c(0, 15, 0, 0)))

)

dev.off()

pdf(file=paste(output_dir, "\\ROC_5_year_trainging.pdf", sep = ""),width=6,height=6)

ggplot(data = time_ROC_df) +

geom_line(aes(x = FP_5year, y = TP_5year), size = 1, color = "#E18727FF") +

geom_abline(slope = 1, intercept = 0, color = "grey", size = 1, linetype = 2) +

theme_bw() +

annotate("text",

x = 0.75, y = 0.15, size = 4.5,

label = paste0("AUC at 5 years = ", sprintf("%.3f", time_roc_res$AUC[[3]])), color = "#E18727FF"

) +

labs(x = "False positive rate", y = "True positive rate") +

theme(

axis.text = element_text(face = "bold", size = 11, color = "black"),

axis.title.x = element_text(face = "bold", size = 14, color = "black", margin = margin(c(15, 0, 0, 0))),

axis.title.y = element_text(face = "bold", size = 14, color = "black", margin = margin(c(0, 15, 0, 0)))

)

dev.off()

pred_f_validation<-predict(f_cph,validation_dataset,type="lp")#!!!type="lp",

data_table<-data.frame(time=validation_dataset[,"OS"],status=validation_dataset[,"Censor"],score=pred_f_validation)

time_roc_res <- timeROC(

T = data_table$time,

delta = data_table$status,

marker = data_table$score,

cause = 1,

weighting="marginal",

times = c(12, 3*12, 5*12),

ROC = TRUE,

iid = TRUE

)

time_ROC_df <- data.frame(

TP_1year = time_roc_res$TP[, 1],

FP_1year = time_roc_res$FP[, 1],

TP_3year = time_roc_res$TP[, 2],

FP_3year = time_roc_res$FP[, 2],

TP_5year = time_roc_res$TP[, 3],

FP_5year = time_roc_res$FP[, 3]

)

pdf(file=paste(output_dir, "\\ROC_135_years_validation.pdf", sep = ""),width=6,height=6)

ggplot(data = time_ROC_df) +

geom_line(aes(x = FP_1year, y = TP_1year), size = 1, color = "#BC3C29FF") +

geom_line(aes(x = FP_3year, y = TP_3year), size = 1, color = "#0072B5FF") +

geom_line(aes(x = FP_5year, y = TP_5year), size = 1, color = "#E18727FF") +

geom_abline(slope = 1, intercept = 0, color = "grey", size = 1, linetype = 2) +

theme_bw() +

annotate("text",

x = 0.75, y = 0.25, size = 4.5,

label = paste0("AUC at 1 year = ", sprintf("%.3f", time_roc_res$AUC[[1]])), color = "#BC3C29FF"

) +

annotate("text",

x = 0.75, y = 0.15, size = 4.5,

label = paste0("AUC at 3 years = ", sprintf("%.3f", time_roc_res$AUC[[2]])), color = "#0072B5FF"

) +

annotate("text",

x = 0.75, y = 0.05, size = 4.5,

label = paste0("AUC at 5 years = ", sprintf("%.3f", time_roc_res$AUC[[3]])), color = "#E18727FF"

) +

labs(x = "False positive rate", y = "True positive rate") +

theme(

axis.text = element_text(face = "bold", size = 11, color = "black"),

axis.title.x = element_text(face = "bold", size = 14, color = "black", margin = margin(c(15, 0, 0, 0))),

axis.title.y = element_text(face = "bold", size = 14, color = "black", margin = margin(c(0, 15, 0, 0)))

)

dev.off()

f_cph_1 <- cph(Surv(OS,Censor) ~ Age+Stage+Risk_level,

x=T, y=T, surv=T,

data=training_dataset)

cal_1<-calibrate(f_cph_1,u=365,cmethod='KM',m=80,B=300)# usually B=200 or 300

#par(mar=c(7,4,4,3),cex=1.0)

pdf(file=paste(output_dir, "\\calibrate_1_years_training.pdf", sep = ""),width=6,height=6)

plot(cal_1,lwd=2,lty=1, ##设置线条形状和尺寸

errbar.col=c(rgb(0,118,192,maxColorValue = 255)),

xlab='Nomogram-Predicted Probability of 1 years OS',

ylab='Actual 1 years OS(proportion)',

col=c(rgb(192,98,83,maxColorValue = 255)),

xlim = c(0,1),ylim = c(0,1),

mgp = c(2, 1, 0))

dev.off()

f_cph_2 <- cph(Surv(OS,Censor) ~ Age+Stage+Risk_level,

x=T, y=T, surv=T,

data=training_dataset)

cal_2<-calibrate(f_cph_2,u=365*3,cmethod='KM',m=80,B=300)# usually B=200 or 300

#plot(cal_2)

pdf(file=paste(output_dir, "\\calibrate_3_years_training.pdf", sep = ""),width=6,height=6)

plot(cal_2,lwd=2,lty=1,

errbar.col=c(rgb(0,118,192,maxColorValue = 255)),

xlab='Nomogram-Predicted Probability of 3 years OS',

ylab='Actual 3 years OS(proportion)',

col=c(rgb(192,98,83,maxColorValue = 255)),

xlim = c(0,1),ylim = c(0,1),

mgp = c(2, 1, 0))

dev.off()

f_cph_3 <- cph(Surv(OS,Censor) ~ Age+Stage+Risk_level,

x=T, y=T, surv=T,

data=training_dataset)

cal_3<-calibrate(f_cph_3,u=365*5,cmethod='KM',m=80,B=300)# usually B=200 or 300

pdf(file=paste(output_dir, "\\calibrate_5_years_training.pdf", sep = ""),width=6,height=6)

plot(cal_3,lwd=2,lty=1,

errbar.col=c(rgb(0,118,192,maxColorValue = 255)),

xlab='Nomogram-Predicted Probability of 5 years OS',

ylab='Actual 5 years OS(proportion)',

col=c(rgb(192,98,83,maxColorValue = 255)),

xlim = c(0,1),ylim = c(0,1),

mgp = c(2, 1, 0)) dev.off()
