# Supplementary material for: Development of a necroptosis-related prognostic model for uterine corpus endometrial carcinoma
Source: Sci Rep. 2024 Feb 21;14:4257. doi: 10.1038/s41598-024-54651-3 (PMC10881509; doi:10.1038/s41598-024-54651-3)

Supplementary Table 1. List of 119 intersect target genes for hsa-miR-425-5p and hsa-miR-7-5p in miRDB, TargetScan and miRTarBase databases.

| Intersect genes of three databases |
| --- |
| hsa-miR-29a-3pFOXJ3  BCOR  MAP3K5  PRDM8  TRMT13  PDCD10  MAP2K6  DNAJC27  AP3M1  SYNCRIP  AFF4  PPP2CB  SERP1  THRB  RNF168  LCOR  HDLBP  FOXN3  TGFB1  PURB  CREBZF  RNF11  ARIH1  ZNF148  PITPNC1  YOD1  PTAR1  DPYSL2  SPATA2  POLE4  RBMS3  UBXN2B  KLF4  PIK3CD  CNN3  PARP1  C5orf22  CHAMP1  ATXN1  IDE  RAF1  CKAP4  FNDC4  ZC3H4  NREP  CACNG7  IRS2  PDE4D  RYK  PSME3  IRS1  KIF16B  PFN2  RRAS2  PIGH  GATA6  LRRC59  SNCA  ARF4  GJC1  ZBTB22  PLP2  RSPRY1  RAB11FIP5  GALNT3  NXT2  SPTY2D1  OSBPL11  UBQLN4  SLC6A9  EGFR  VPS26A  ZNF805  EXOSC2  VDAC1  EIF4EBP2  SMARCD1  PIK3R3  NDFIP2  ADCY9  ASXL1  SOCS2  SLC5A3  C1orf21  TFRC  SQSTM1  PLEC  EHD1  CNOT8  CUL5  RAD54L2  CALU  CRLS1  PPIF  SLC25A15  PAX6  LSM12  NIPAL3  TNRC6B  NDUFA4  ZFAND4  EIF4E  CAMK2D  POGK  NR1H2  HAP1  CCNT2  ALG9  CAPZA1  SRGAP2  GATAD2B  BMPR2  SMAD2  DNAJC5  AKT3  FAM126B  TNRC6A  IGSF8  RCC2 |

Supplementary Table 2. List of 11 immune related intersect genes for hsa-miR-425-5p and hsa-miR-7-5p in miRDB, TargetScan and miRTarBase databases.

| Immune related intersect genes |
| --- |
| THRB  TGFB1  PIK3CD  RAF1  PSME3  EGFR  PIK3R3  TFRC  NR1H2  BMPR2  AKT3 |

Supplementary Figure 1. The Unedited Original Western Blot Image of THRB in UCEC samples.


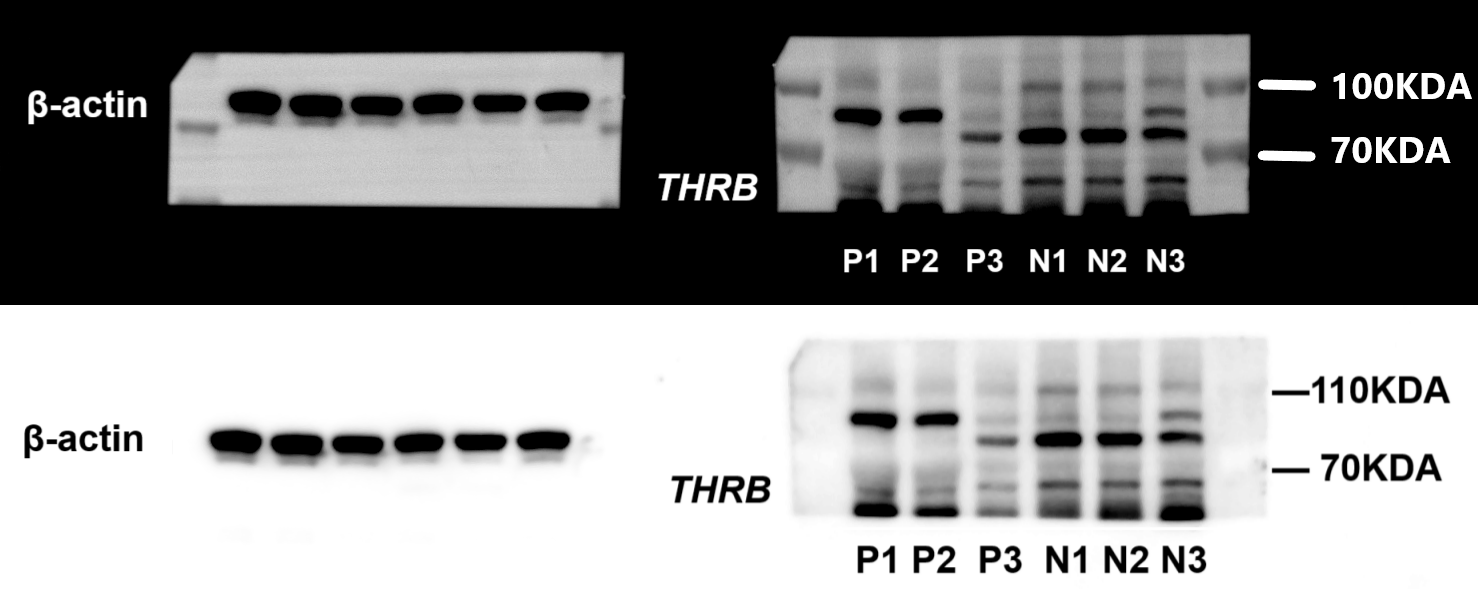

Supplement: Supplementary file 2 — Supplementary Information 2. [file 41598_2024_54651_MOESM2_ESM.docx]
