# Supplementary material for: Development of a necroptosis-related prognostic model for uterine corpus endometrial carcinoma
Source: Sci Rep. 2024 Feb 21;14:4257. doi: 10.1038/s41598-024-54651-3 (PMC10881509; doi:10.1038/s41598-024-54651-3)

Original images of the WB.


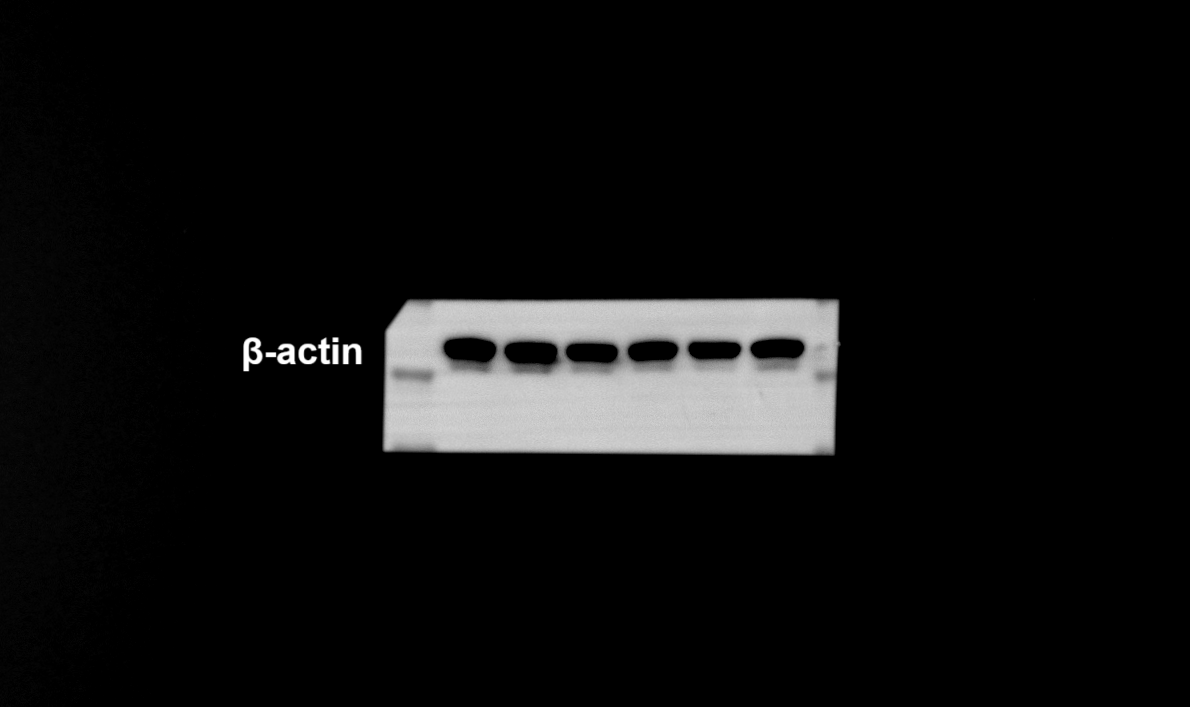

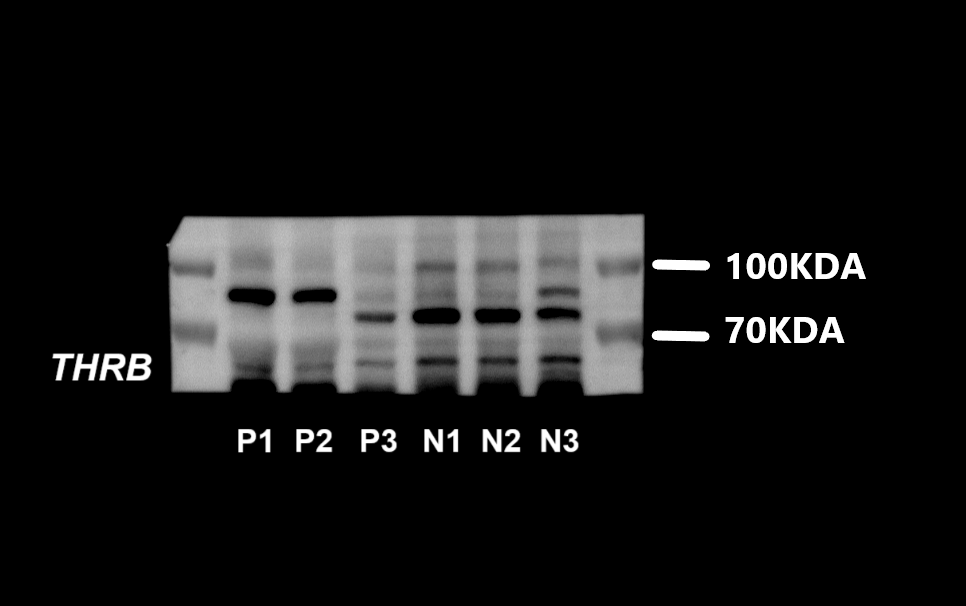

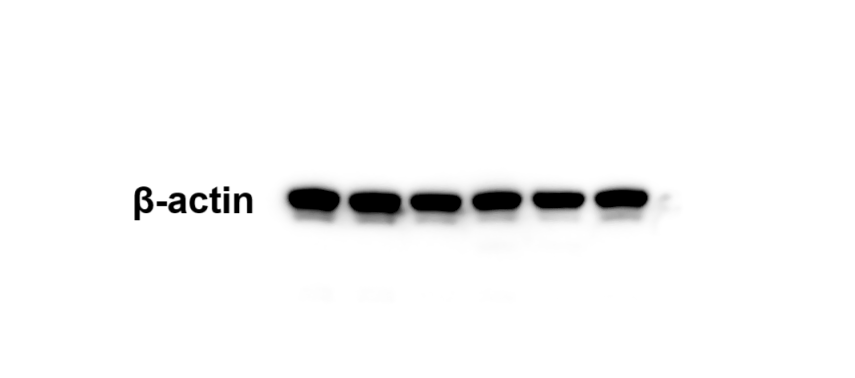

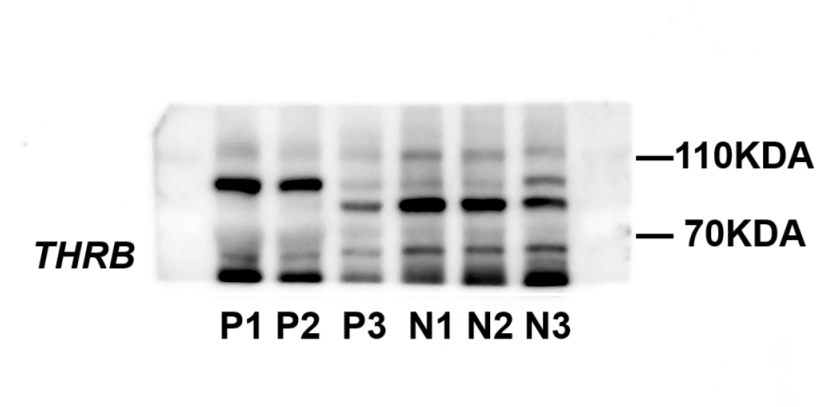


Quantitative analysis of THRB (gray value).


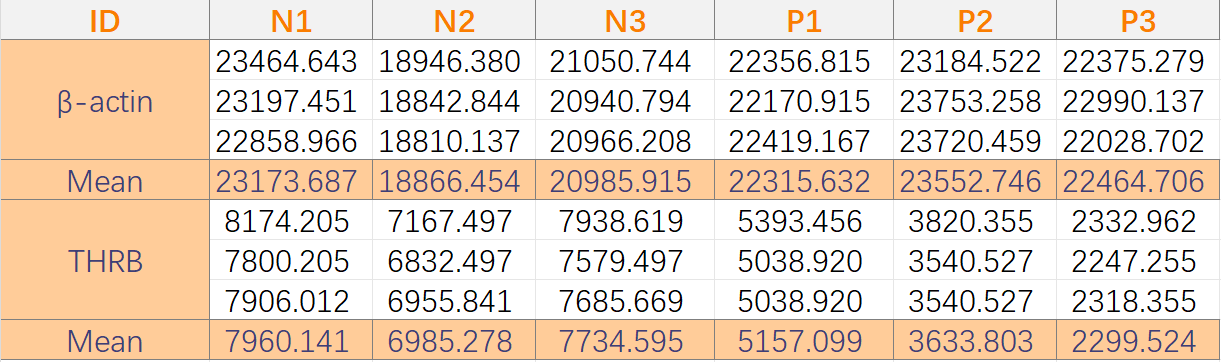


Relative expression levels of THRB.


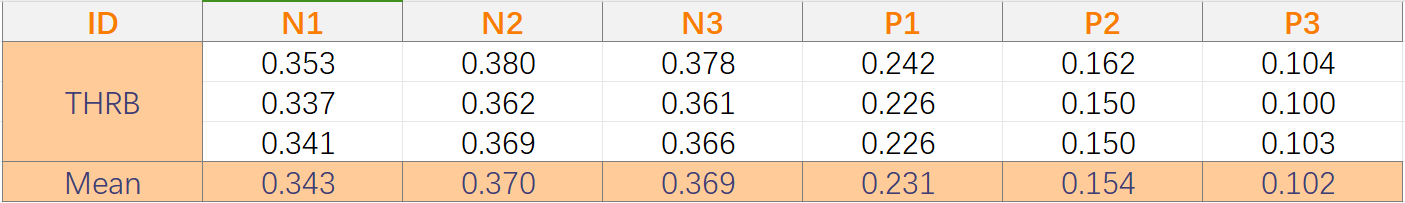

Supplement: Supplementary file 3 — Supplementary Information 3. [file 41598_2024_54651_MOESM3_ESM.docx]
